# Insights into SARS‐CoV‐2 in Angola during the COVID‐19 peak: Molecular epidemiology and genome surveillance

**DOI:** 10.1111/irv.13198

**Published:** 2023-09-22

**Authors:** Ngiambudulu M. Francisco, Stephanie van Wyk, Monika Moir, James Emmanuel San, Cruz S. Sebastião, Houriiyah Tegally, Joicymara Xavier, Akhil Maharaj, Zoraima Neto, Pedro Afonso, Domingos Jandondo, Joana Paixão, Julio Miranda, Kumbelembe David, Luzia Inglês, Amilton Pereira, Agostinho Paulo, Raisa Rivas Carralero, Helga Reis Freitas, Franco Mufinda, Silvia Lutucuta, Mahan Ghafari, Marta Giovanetti, Jennifer Giandhari, Sureshnee Pillay, Yeshnee Naidoo, Lavanya Singh, Derek Tshiabuila, Darren Patrick Martin, Lucious Chabuka, Wonderful Choga, Dorcas Wanjohi, Sarah Mwangi, Yusasha Pillay, Yenew Kebede, Edwin Shumba, Pascale Ondoa, Cheryl Baxter, Eduan Wilkinson, Sofonias Kifle Tessema, Aris Katzourakis, Richard Lessells, Tulio de Oliveira, Joana Morais

**Affiliations:** ^1^ Grupo de Investigação Microbiana e Imunológica Instituto Nacional de Investigação em Saúde Luanda Angola; ^2^ Center for Epidemic Response and Innovation (CERI), School of Data Science and Computational Thinking Stellenbosch University Stellenbosch South Africa; ^3^ KwaZulu‐Natal Research Innovation and Sequencing Platform (KRISP), Nelson R Mandela School of Medicine University of KwaZulu‐Natal Durban South Africa; ^4^ Centro de Investigação em Saúde de Angola (CISA) Caxito Angola; ^5^ Ministério da Saúde Luanda Angola; ^6^ Direcção Nacional de Saúde Pública Ministério da Saúde Luanda Angola; ^7^ Reference Laboratory of Flavivirus Oswaldo Cruz Foundation Rio de Janeiro Brazil; ^8^ Division of Computational Biology, Department of Integrative Biomedical Sciences University of Cape Town Cape Town South Africa; ^9^ Institute of Infectious Disease and Molecular Medicine (IDM), Faculty of Health Sciences, University of Cape Town Cape Town South Africa; ^10^ Africa CDC Institute of Pathogen Genomics Africa Centre for Disease Control and Prevention Addis Ababa Ethiopia; ^11^ African Society for Laboratory Medicine Addis Ababa Ethiopia; ^12^ Department of Biology Oxford University Oxford UK; ^13^ Big Data Institute, Nuffield Department of Medicine University of Oxford Oxford UK

**Keywords:** Angola, COVID‐19, genomic surveillance, SARS‐CoV‐2, variants of concern

## Abstract

**Background:**

In Angola, COVID‐19 cases have been reported in all provinces, resulting in >105,000 cases and >1900 deaths. However, no detailed genomic surveillance into the introduction and spread of the SARS‐CoV‐2 virus has been conducted in Angola. We aimed to investigate the emergence and epidemic progression during the peak of the COVID‐19 pandemic in Angola.

**Methods:**

We generated 1210 whole‐genome SARS‐CoV‐2 sequences, contributing West African data to the global context, that were phylogenetically compared against global strains. Virus movement events were inferred using ancestral state reconstruction.

**Results:**

The epidemic in Angola was marked by four distinct waves of infection, dominated by 12 virus lineages, including VOCs, VOIs, and the VUM C.16, which was unique to South‐Western Africa and circulated for an extended period within the region. Virus exchanges occurred between Angola and its neighboring countries, and strong links with Brazil and Portugal reflected the historical and cultural ties shared between these countries. The first case likely originated from southern Africa.

**Conclusion:**

A lack of a robust genome surveillance network and strong dependence on out‐of‐country sequencing limit real‐time data generation to achieve timely disease outbreak responses, which remains of the utmost importance to mitigate future disease outbreaks in Angola.

## 1 INTRODUCTION

The COVID‐19 disease caused by the SARS‐CoV‐2 virus has infected over 600 million (M) people and resulted in more than 6.8 M deaths (as of May 2023).^1^ However, Africa accounted for only 12.8 M cases and 259,000 reported deaths.^1^ These reports were likely vastly underestimated as significant excess mortalities were reported throughout Africa.^2^, ^3^ A high estimated incidence of asymptomatic infections and limited testing in many regions^4^ support the notion that many case numbers remain undocumented.^5^ These observations also hold for the Southwestern African country of Angola.^5^

The disease transmission dynamics and the pandemic progression within many African countries, including Angola, are still not investigated in detail. Angola lacks a strong genomic surveillance network, despite the emergence of two variants of concern (VOCs) within the southern African region,^6^, ^7^ and outbreaks of reemerging infectious diseases.^8^, ^9^, ^10^, ^11^ This may hamper the timely detection and early warning of newly emerging VOCs, variants of interest (VOIs), and other outbreaks of infectious diseases of global importance.

Angola, the largest and wealthiest of the Portuguese‐speaking African states, has a population of approximately 35 M residents.^1^ Despite its significant gross domestic product, the country has been grappling with inequality and post‐independence armed conflict, which have negatively impacted its economy. As a result, crucial national sectors such as healthcare and disease surveillance networks become constrained. This lack of infrastructure is especially concerning during infectious disease outbreaks, where the robustness of the healthcare system and pathogen surveillance initiatives are heavily dependent on it to facilitate effective disease control and prevention.^6^, ^7^, ^8^, ^9^, ^10^

Using SARS‐CoV‐2 sequences generated for genomic surveillance in Angola during the peak of the COVID‐19 pandemic, we investigated the pandemic progression and disease dynamics for the first 27 months of the pandemic. In this study, we investigated the transmission of viruses within communities and across borders. We were interested in identifying novel SARS‐CoV‐2 lineages and variants circulating in Angola. In addition, we sought to determine the timing and source of virus introductions into Angola and the larger southern‐central African region. Genomic surveillance capacity must be developed within Angola to provide valuable insights into circulating variants and inform effective strategies to mitigate COVID‐19 and other emerging or re‐emerging pandemics.

## 2 METHODS

### 2.1 Ethics

The study received both Health Research Authority approval (Instituto Nacional de Investigação em Saúde, Angola) and ethical approval (Ministry of Health Ethics Committee, Angola, Number: 27/C.E/2021). The use of South African samples for sequencing and genomic surveillance was approved by the University of KwaZulu‐Natal Biomedical Research Ethics Committee (ref. BREC/00001510/2020), the University of the Witwatersrand Human Research Ethics Committee (HREC) (ref. M180832), Stellenbosch University HREC (ref. N20/04/008_COVID‐19), the University of the Free State Research Ethics Committee (ref. UFS‐HSD2020/1860/2710), and the University of Cape Town HREC (ref. 383/2020).

### 2.2 SARS‐CoV‐2 samples and metadata

We obtained anonymous nasopharyngeal and oropharyngeal swab samples from patients that tested positive for SARS‐CoV‐2. RT‐qPCR analyses were conducted at the Instituto Nacional de Investigação em Saúde, in Angola.

### 2.3 Real‐time PCR

RNA was extracted using the MagMAX™ Virus/Pathogen II Nucleic Acid Isolation Kit (Cat. No. A48383) using the automated nucleic acid extractor KingFisher™ Apex Magnetic Particle Processor. The protocol was completed as described previously.^7^ The RT‐PCR analyses were performed using the TaqPath COVID‐19 CE‐IVD RT‐PCR Kit (Life Technologies, Carlsbad, CA). Experimental procedures were applied according to the manufacturer's instructions. Samples submitted for downstream analyses were delineated based on cycle threshold (Ct) values, where Ct values <30 were considered sufficient for this study. These samples were subjected to SARS‐CoV‐2 whole genome sequencing (WGS).

### 2.4 Whole‐genome sequencing and assembly

All samples for WGS were sequenced and assembled at out‐of‐country facilities, namely, the Centre of Epidemic Response and Innovation (CERI), Stellenbosch University, South Africa, and Kwazulu‐Natal Research Innovation and Sequencing Platform (KRISP), University of KwaZulu‐Natal, South Africa. WGS samples were subjected to RNA extraction using the Chemagic 360 instrument and the CMG‐1033‐S kit (Perkin Elmer, Hamburg, Germany). The experimental procedures followed the manufacturer's instructions. Isolated RNA was stored at −80°C. Complementary DNA (cDNA) synthesis was implemented as described previously.^8^ The multiplex PCR was performed according to the nCoV‐2019 ARTIC network sequencing (https://artic.network/ncov-2019) or the Midnight sequencing protocol^9^ with the V3 and V4 primer sets. The PCR products were purified using the AmpureXP purification beads (Beckman Coulter, High Wycombe, UK). The purified PCR products were quantified using the Qubit 4.0 instrument (Life Technologies Carlsbad, CA) using the Qubit dsDNA High Sensitivity assay. Indexed paired‐end libraries were prepared using the DNA Illumina® Nextera Flex DNA Library Prep kit as described previously.^8^ The resulting libraries were normalized to 2 nM, pooled and denatured with 0.2 N sodium hydroxide. Depending on reagent availability, either Illumina or Oxford Nanopore whole genome sequencing was performed. For Illumina sequencing, PhiX Control v3 adapter‐ligated library was used as a control, and the 1.3 pM sample library was spiked with 1% PhiX. Libraries were loaded onto a 300‐cycle NextSeq 500/550 Mid Output Kit v2, on the Illumina NextSeq550 instrument (Illumina, San Diego, CA, USA), or using an SQK‐RBK 110‐96 kit on the GridION Mk (Oxford Nanopore Technologies, Oxford, UK).

### 2.5 Sequence assembly and quality control

The raw reads were assembled using Genome Detective Coronavirus Typing Tool (version 1.133).^10^ Genome Detective reports and those generated using Nextclade^11^ were used to evaluate the quality of the assemblies. Assembled genomes were mapped to the Wuhan‐Hu‐1 reference NC_045512.2 (accession number: MN908947.3) and subjected to manual curation to remove low‐quality mutations and sequencing and assembly errors using Geneious Prime v2021.2.2. To do so, we accessed BAM files generated using Genome Detective and resolved mutations flagged by the Nextclade assembly reports. Genome assemblies of sufficient quality (genome coverage >80%) were deposited to GISAID (EPI_SET_230222vy and DOI: 10.55876/gis8.230222vy)^12^ (https://www.gisaid.org/).

### 2.6 Epidemiological data

The COVID‐19 Data Repository by the Centre for Systems Science and Engineering at John Hopkins University was accessed through Our World In Data (OWID; https://ourworldindata.org/ ^1^) and included data generated from January 1, 2020, to April 22, 2022. The daily estimates on the effective reproductive number (Median Rₑ with 95% highest posterior density [HPD] intervals) were retrieved from COVID‐19 Rₑ (https://github.com/covid-19-Re/dailyRe-Data). Rₑ was used as an indication of the average number of secondary infections acquired from an infected person and served as a basis to estimate the extent and progression of the COVID‐19 pandemic. For these analyses, an Rₑ value >1 represented a growth in the epidemic, and Rₑ values <1 were used to indicate a reduction in the epidemic progression.

Genome assemblies and metadata were obtained from GISAID (https://www.gisaid.org/)^12^ (accessed on April 22, 2022). For these analyses, 1201 genome assemblies generated from SARS‐CoV‐2 samples originating from Angola with available and complete metadata were plotted per province to illustrate the spatial distribution of genomic surveillance across the country. For comparative purposes, genome data generated from samples originating from African countries were included in these analyses. Changes in the frequency of the genetic composition of SARS‐CoV‐2 and its variant lineages were visualized by plotting the genome count per lineage over the date of sampling. Figures were generated using R Studio (RStudio Team 2020). The pandemic progression in Angola was illustrated using Timeline Storyteller.^13^

### 2.7 Phylogenetic analyses

To infer the phylogenetic relationship between SARS‐CoV‐2 genomes sampled from Angola and those generated from neighboring countries, we included sequences generated from global and South‐Central African regions, thereby reflecting global and South‐central African virus diversity. Due to the importance of the C.16 and B.1.1.275 lineages in the South‐central region of Africa, we included all available C.16 (*n* = 74) and B.1.1.275 (*n* = 70) sequences from neighboring countries. The dataset consisted of 18,704 genome sequences.

The resulting dataset was subjected to whole‐genome alignment implemented using NextAlign.^11^ To ensure accurate codon alignment, the alignment was curated manually using Geneious Prime software version 2021.1.1. The Tree topology was determined using maximum likelihood (ML) analyses implemented in IQTree.^14^ To infer the branching structure in the SARS‐CoV‐2 phylogenetic tree, a general time reversible model of nucleotide substitution was implemented on 1000 bootstrap replicates. The resulting tree was rooted using the Wuhan reference strain and annotated using the *GGTREE* package implemented using R.^15^

Due to the divergence between Omicron and non‐Omicron sequences, the ML‐tree topology could not be dated using TreeTime.^16^ To address this, we split the dataset into Omicron (4291 taxa including 39 Angolan) and non‐Omicron (15 617 taxa including 1165 Angolan) subsets for dating and discrete ancestral state reconstruction as previously described.^8^

The resulting tree topologies were transformed into time‐calibrated trees with the TreeTime package, and a fixed mutation rate was determined by regression of root‐to‐tip diversity in each subset using the clock functionality of TreeTime. Possible outlier sequences that violate the strict molecular clock assumption were pruned from the ML tree topologies with the *APE* package implemented in R. The dated phylogenies along with associated metadata were mapped to discrete country locations, inferring country locations for internal nodes in the trees using the *Mugration* package extension in TreeTime.^16^

A custom Python script was utilized to estimate dates of viral exchange events occurring between Angola and other countries. For these analyses, the total count of discrete character changes in the phylogeny was investigated from the tree root toward the tips. These analyses were repeated on 10 bootstrap replicate trees that were chosen at random to assess possible variation in viral exchanges. The results were illustrated using a bar chart and chord plots generated using R.

### 2.8 Phylogeographic reconstruction

Monophyletic clusters were identified using the Phylotype^17^ (size ≥5, size/different ≥1, persistence ≥1, nodes which ML, bootstrap values were ≥70 were considered, tested for ACCTRAN optimization, and 1000 iterations of shuffling procedures). The C.16 lineage yielded the most extensive and only significant monophyletic cluster with 216 taxa. For these, we modelled the phylogenetic diffusion and spread by analyzing localized transmission (between neighboring countries) using a continuous phylogeography implemented in BEAST v1.10.4 under a strict molecular clock assumption and the exponential growth coalescent model as previously described by Sagulenko and colleagues.^16^ For each sequence, latitude and longitude coordinates were attributed to the lowest administrative level location specified in the GISAID metadata. Markov chain Monte Carlo (MCMC) analyses were set up in BEAST v1.10.4 in duplicate for 50 million interactions and sampling every 5000 steps in the chain. Convergence was assessed in Tracer v1.7.1 (ESS for all relevant model parameters >200).^18^ Maximum clade credibility trees for each run were summarized using *TreeAnnotator* after discarding the initial 10% as burn‐in. We used the R package “seraphim”^19^ to extract and map spatiotemporal information embedded in the posterior trees. A transmission link on the phylogeographic map can denote one or more transmission events depending on the phylogeographic inference.

### 2.9 Data availability statement

The SARS‐CoV‐2 whole genome sequences that were investigated were retrieved from the GISAID sequence database. A full list of the Angolan sequences as well as global references is presented and acknowledged using the GISAID EPI set identifier EPI_SET_230222vy and doi:10.55876/gis8.230222vy and/or doi:10.55876/gis8.230222vy and is also available on the following GitHub repository (https://github.com/CERI-KRISP/Molecular-epidemiology-of-SARS-CoV-2-in-Angola). The GitHub repository contains the alignments, raw and time‐scaled ML tree topologies, data analysis and visualization scripts, and all BEAST XML files used here, which will allow for the independent reproduction of the results.

## 3 RESULTS

### 3.1 Pandemic progression and milestones in Angola

**FIGURE 1:**
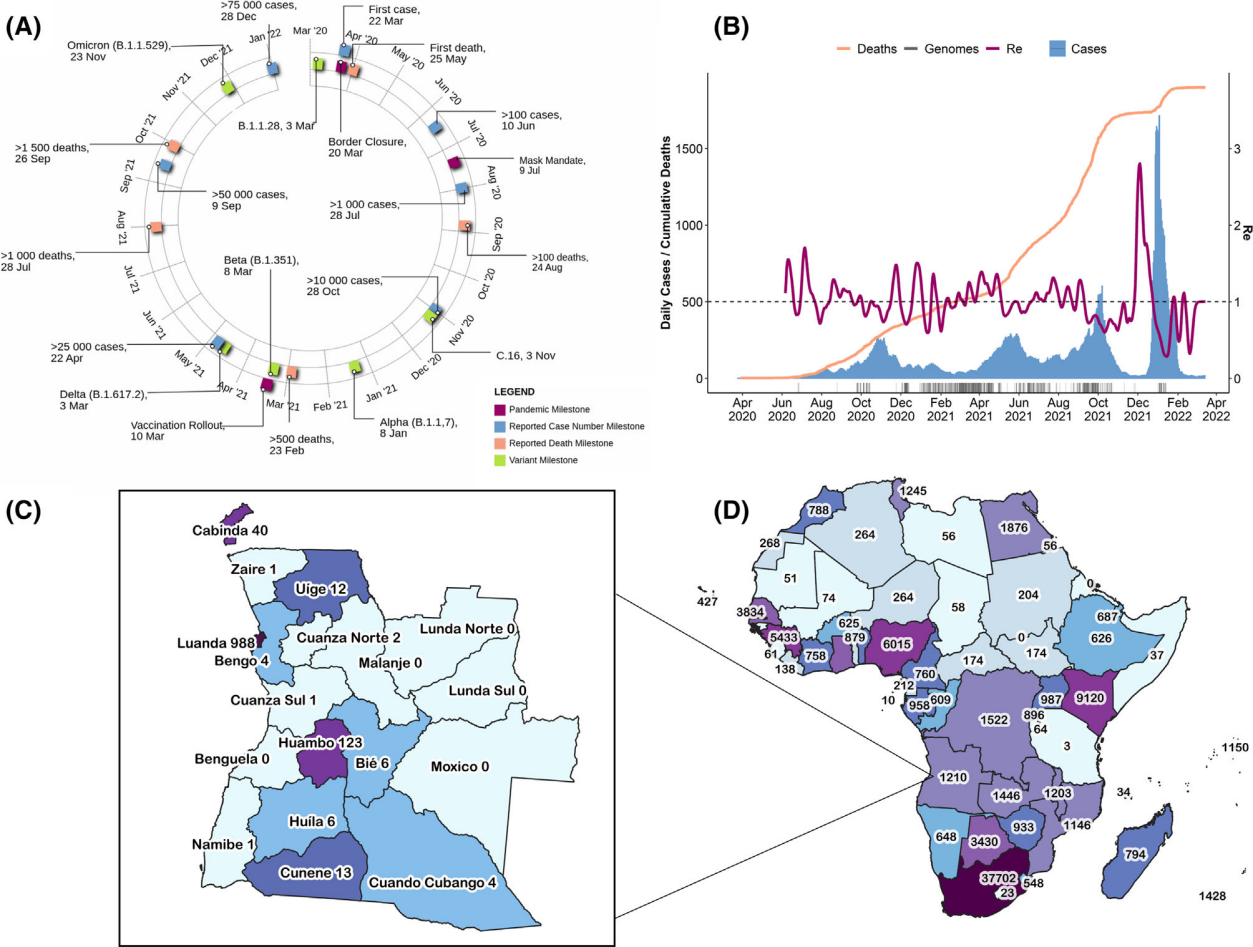
Depiction of epidemiological data and genome sequence for the Republic of Angola from March 2020 to March 2022. (A) Timeline of the pandemic milestones for the Republic of Angola. (B) Epidemiological curve illustrating daily recorded COVID‐19 cases (illustrated in blue; y‐axis), the cumulative number of reported deaths (orange; y‐axis), the effective reproductive number R_e_ (pink, alternative y‐axis), and number of sequenced genomes from the recorded date of sampling. (C) A schematic representation of the total count of sequenced genomes per province of the Republic of Angola and (D) for the African continent.

The first case of COVID‐19 was reported on March 21, 2020, from an infected traveler returning from Portugal (Figure 1A).^1^ On the same day, travel restrictions and border closures were instated. Following the first reported case, the National Contingency Plan to Manage the Pandemic was instated.^20^ This enabled the Angolan public health response team to increase COVID‐19 testing capacity and implement contact tracing and tracking to curb infections.

A national state of emergency was declared on March 27, 2020, until May 26, 2020, which included a 2‐week national lockdown.^6^ This social and economic lockdown required the suspension of public transportation, and non‐essential businesses were closed. The government implemented measures to mitigate the impact of the lockdown on economically vulnerable groups, such as providing food assistance and grant funding. The lockdown was lifted on May 11, 2020, but several restrictions remained in place to limit the spread of the virus. These included the mandatory usage of facemasks and social distancing in all public settings, restriction on public gatherings, restricted international travel, as well as enforcement of a curfew in some provinces such as Luanda, Benguela, Huíla, and Cuanza Sul, which restricted movement between certain hours of the day.

The vaccination campaign named the COVAX initiative utilizing the Oxford/AstraZeneca vaccine was initiated on March 10, 2021.^6^ Vaccination sites included hospitals, health centers, and public spaces. As part of the vaccination initiative, a mobile vaccination program was launched to bring vaccines to more remote and hard‐to‐reach areas. Initial vaccination strategies prioritized front‐line workers, healthcare workers, and other high‐risk groups.

Following the global trend, despite the implementation of pandemic mitigation strategies, Angola remained vulnerable to viral introductions, and several of the VOIs and VOCs were detected and circulated within the country (Figure 1A). This included the C.16 VOI strain (November 3, 2020), as well as the VOCs Alpha (January 8, 2021), Beta (March 8, 2021), Delta (March 3, 2021), and Omicron (November 23, 2021) strains.

### 3.2 Epidemiological progression of COVID‐19 in Angola

The COVID‐19 pandemic in Angola was defined by four distinct waves of infection during the first 27 months (Figure 1B), resulting in >99,000 cases and ~1900 deaths.^1^ The first wave occurred between mid‐September 2020 and December 2020, resulting in >14,000 cases and ~130 deaths. The second wave of infection occurred between April and August 2021 (~25,000 cases and >1200 deaths) and was shortly followed by a third wave in September and October 2021 (~16,400 cases and >1700 deaths). The fourth, and the most severe, wave in terms of the number of cases per day occurred between December 2021 and January 2022. This wave resulted in >33,500 and ~160 deaths and peaked at 5035 daily cases on December 28, 2021, the highest recorded throughout the pandemic (Figure 1B). The basic reproductive number (R_e_) for the pandemic in Angola follows a cyclical pattern fluctuating around 1 with high R_e_ values preceding major waves of infection, which is followed by lower values as pandemic waves subsided (Figure 1B). The most noteworthy peak occurred directly preceding the fourth wave. Similar to the global trends, the fourth wave, however, was associated with the fewest reported deaths.

### 3.3 SARS CoV‐2 genomes and lineages

In this study, 1204 SARS‐CoV‐2 samples were isolated in Angola that produced whole genome sequences (Figure 1C). Of these sequences, the majority was isolated during February, March, and July of 2021. Most sequences were isolated from Luanda (*n* = 988) and Huambo (*n* = 123); however, no isolates were sampled from Eastern provinces such as Lunda Norte, Lunda Sul, Moxico, and the western province, Benguela. Genome surveillance in Angola generated more data in comparison to its neighboring countries, Namibia (*n* = 648) and the Republic of the Congo (n = 609), but fewer than the neighboring Democratic Republic of Congo (DRC) (*n* = 1522) and Zambia (*n* = 1446) (Figure 1D).

**FIGURE 2:**
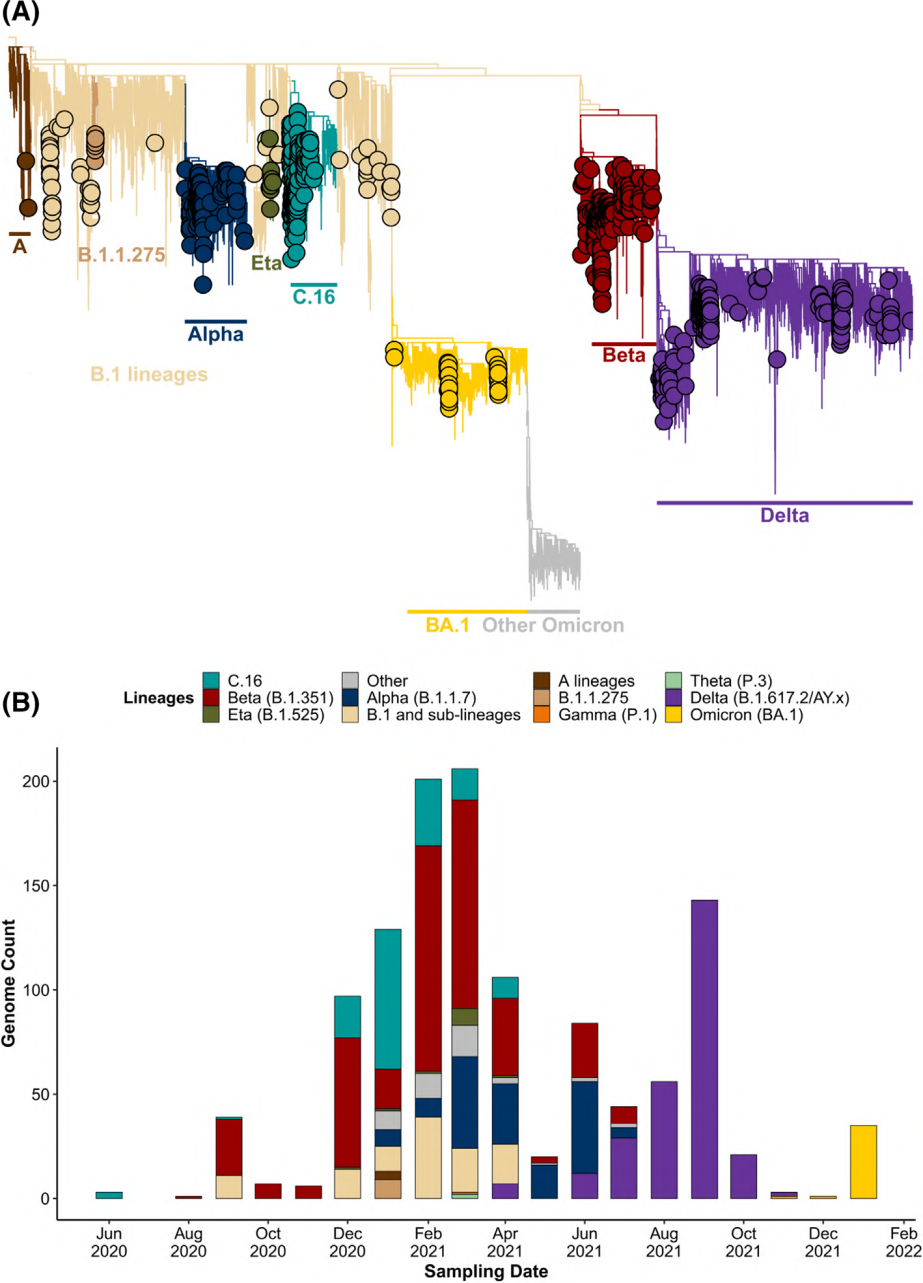
(A) The maximum likelihood phylogenetic tree displays the SARS‐CoV‐2 lineages of interest sequenced from Angolan samples (Angolan genomes were illustrated as circles in the tree). (B) Plot illustrating count of total monthly SARS‐CoV‐2 genomes sequenced per lineage for Angola throughout the epidemic from June 2020 to February 2022.

Analyses of the sequences sampled from Angola indicated an admixture of co‐circulating viruses during the first 27 months of the pandemic. The first of these sequences, which was generated in June 2020, was assigned to the C.16 lineage. This lineage remained prevalent in sampled isolates until April 2021. In August 2020, The Beta strain was the most prevalent variant. Beta remained prevalent in sampled sequences until July 2021 when it was displaced by the Delta strain. Comparable to trends in other African countries,^21^ the B.1 and related sublineages were prevalent during September 2020 and remained prevalent in samples isolated from December 2020 to April 2021. The highest viral diversity was observed from January to March 2021 (Figures 2 and S1). The Alpha variant was sampled from January to July 2021. The VOIs Eta and Theta (December 2020 to April 2021 and January 2021, respectively), the VOC Gamma (January 2021), and other variants, including the A variants (January 2021), were also circulating in Angolan populations. The Omicron variant (B.1) displaced other variants circulating at the time (December 2021), and lineages remained the dominating variant during December 2021 and January 2022.

Interestingly, Angola's first two infection waves were not dominated by single variants. For example, the second wave consisted of the Beta strain, comparable to other southern African countries. However, during this time, the B.1 and descendant lineages, Alpha, and C.16 were also prevalent.

### 3.4 Molecular and phylogenetic analyses of SARS‐CoV‐2 in the Republic of Angola

Molecular state reconstruction inferred multiple and independent introductions of SARS‐CoV‐2 into Angola (Figure 2A,B). These included two separate introductions of the A lineages, and multiple introductions were coupled with community transmission of the Alpha, C.16, B.1 lineages, Beta, Delta, and Eta variants. The Omicron BA.1 variant was the predominant lineage detected in accordance with the global trend of community transmission from December 2021. By February 2022, 100% of the detected cases in the population was all attributed to the BA.1 lineage (Figures 2 and S1).

Of the 1204 Angolan whole‐genome sequences, 147 (12.2%) were assigned to the C.16 lineage according to the Pango classification system. These sequences were isolated from Luanda, from 2020/06/26 to 2021/04/22. Among other African countries, C.16 was detected in Namibia (*n* = 80), South Africa (*n* = 3), Zambia (*n* = 2), Kenya (*n* = 1), Guinea (*n* = 2), DRC (*n* = 7), and Cote d'Ivoire (*n* = 1).^12^ Globally, a total count of 1030 C.16 genomes was deposited on GISAID. Despite these relatively low numbers, the C.16 lineage circulated globally and was detected in Asian countries and cities, such as Abu Dhabi, Japan, Israel, Jordan, and Bahrain, throughout Europe and North America, and a single isolate was retrieved from Brazil.^12^

The C.16 variants detected among the Angola sample set had between 10 to 22 amino acid substitutions compared to the Wuhan Human‐1 ancestral strain. All but one sequence reflected a thymine to guanine point substitution mutation in the spike protein, resulting in an L452R mutation (leucine to arginine non‐synonymous mutation), and a D614G mutation at position 23,404 (adenosine to guanine point mutation). The two genetic alterations within the Spike protein were previously reported from B.1.617.1 lineages. The Spike L452R mutation was also prevalent in the VOC Delta. Of the C.16 assemblies, 127 had three consecutive point mutations in the nucleocapsid protein, namely, G2881A, G2882A, and G2883C substitutions that resulted in the R203K and the G204 amino acid substitutions. The C.16 lineage had the orf1a: G3302A, C4002T substitutions, E1013K, and T1246I amino acid substitutions, respectively. This lineage also had a C14408T substitution that resulted in a P3146 amino acid substitution in the orf1b open reading frame.

### 3.5 Introductions of SARS‐CoV‐2 into the Republic of Angola

**FIGURE 3:**
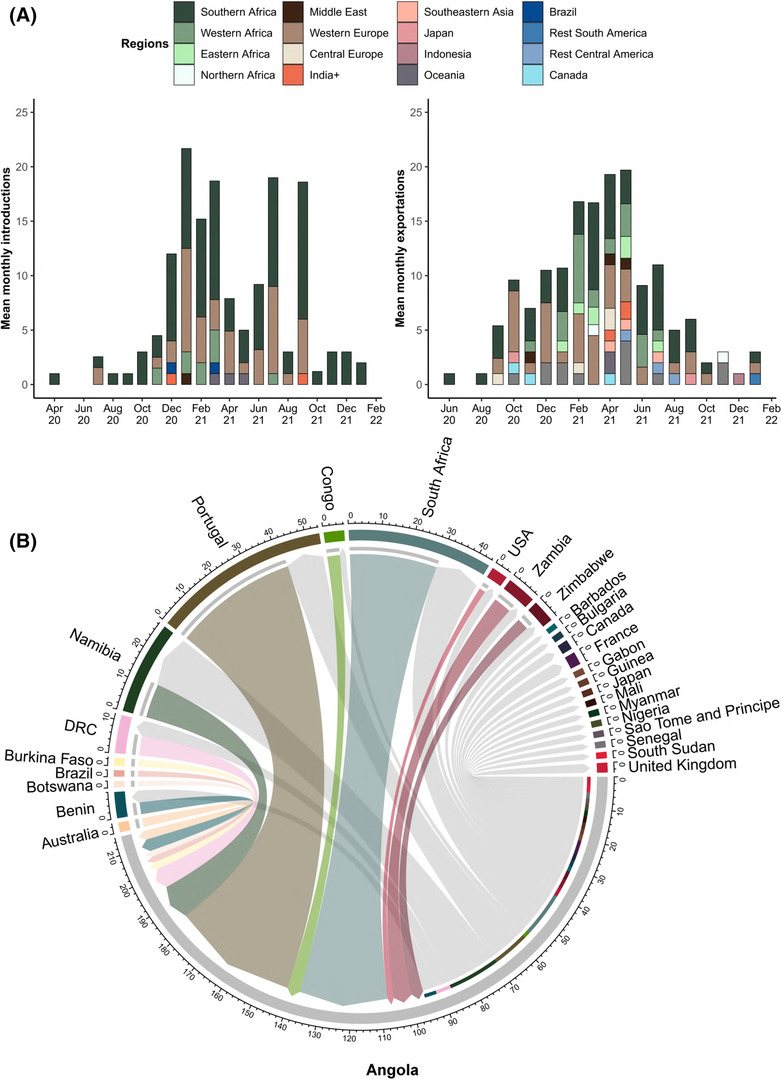
(A) The mean number of sources of SARS‐CoV‐2 virus introductions into the Republic of Angola per global region per month and (B) the mean monthly number of virus exportations from Angola into different regions of the world. For these analyses, “India+” denotes both India and Bangladesh. (C) The chord plot displays the mean virus introductions from each source country into Angola (colored chords), as well as the mean number of virus exportations from Angola into the destination countries (gray chords). The arrowheads display the direction of movement while chord size indicates the number of virus exchanges.

Angola remains vulnerable to virus introduction events from neighboring and international countries (Figure 3A). The first introduction event was inferred in early 2020, and the more significant introduction events were inferred to have resulted from introductions originating from southern African countries such as South Africa; however, other destinations such as West‐ and Eastern Africa, the Middle East, India (and Bangladesh), and Oceania have also served as sources of virus introduction events (Figure 3A). The highest number of inferred introduction events in Angola occurred during January, March, July, and September 2021, and the fewest were May, June, August to October 2020, and October 2021 (Figure S2). Portugal and South Africa had the highest inferred introduction events (Figure S2). However, neighboring countries such as the DRC, Namibia, and Zambia also contributed to the most virus introduction events into Angola (Figure 3C). Angola received the highest count in inferred virus introduction events from Portugal during December 2020, April 2021, and July 2021. Namibia served as a source from April to December 2021, and the highest of these were recorded during July and September 2021 (Figure S2A). Phylogenetic analyses indicated that noteworthy introduction events included the B.1.1.7 strain most likely introduced from Europe during the first days of January 2021, the B.1.351 (502Y.V2) on six separate occasions from South Africa during January 2021, and the B.1.1.275 strains that resulted in an outbreak occurring in an oil rig in 2020.^22^

Phylogenetic inference further indicated that Angola served as a source of viral expiration events in neighboring African and international countries (Figure 3B). Geographical regions such as Southern, Western, Eastern, Central, and Northern Africa, West and Central Europe, South and Central America, the Middle East, Indonesia, Oceania, Japan, Southeastern Asia, Brazil, and Canada were prone to virus exportation events originating from Angola (Figure 3B). The fewest virus exportation events occurred between June–August 2020 and December 2021. The months with the highest mean of exportation events included February to May 2021, exporting to Southern and Western Africa, Western Europe, Oceania, and to a lesser extent India (and Bangladesh), Southeastern Asia, Canada, and the Middle East (Figure S2B). From October 2020 to January 2022, Angola continuously served as a source of SARS‐CoV‐2 to Portugal, with the highest means inferred for December 2020 and April 2021. Similarly, South Africa received viral exchanges continuously between September 2020 to October 2021, and the highest mean in viral exchange occurred during March 2021. Namibia received viral exportation from July 2020 to August 2021, with the highest mean recorded during June 2021 (Figure 3C).

### 3.6 The spread and virus transmission of the C.16 lineage of SARS‐CoV‐2 from Angola to neighboring countries

**FIGURE 4:**
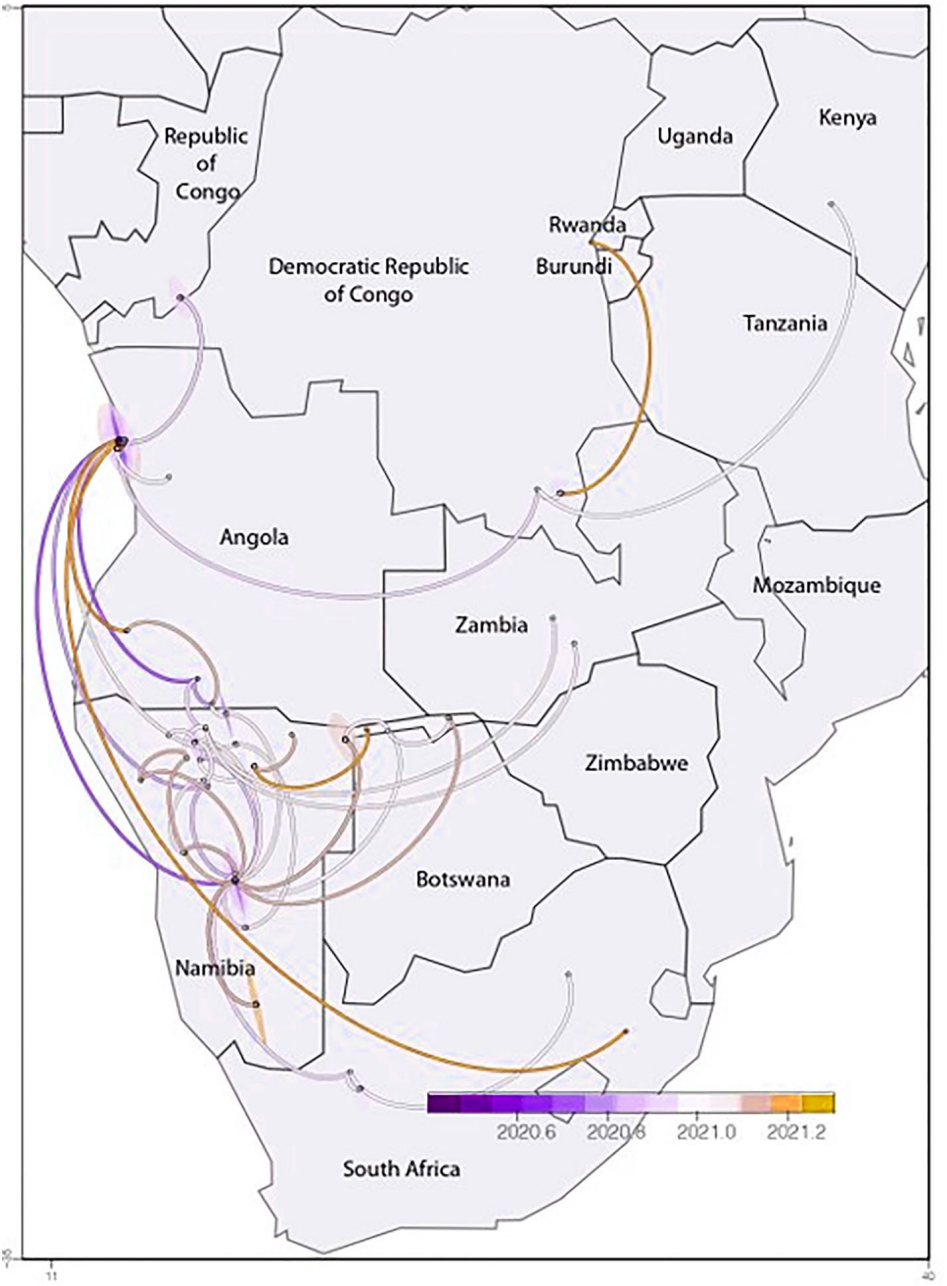
Illustration of the spread and virus transmission events inferred by the phylogeographic reconstruction of the C.16 lineage of SARS‐CoV‐2 from Angola to neighboring countries. The transmission paths were colored according to the inferred dates of transmission.

Due to the persistence of the C.16 lineage in Angola, its evolutionary history and special temporal dispersal were investigated in greater detail. Phylogeographic investigations indicated that the first isolate of C.16 indicated that it emerged in March 2020 in Luanda; however, the first Angolan C.16 lineage was only sampled on June 26, 2020 (Figure 4). Since its emergence, community transmission in Luanda occurred during 2020 and 2021. Phylogeographic reconstruction illustrated that the C.16 lineage was disseminated locally, originating from Luanda, and was disseminated to the cities of Santa Clara in September 2020, Ondjiva in July 2020, N'dalatando in January 2021, and Lubango in February 2021.

Virus movement from Angola to neighboring Namibia was inferred from four independent events (Figure 4). Three of these introductions originated from Luanda in May, June, and November 2020. A single transmission event, which originated from Santa Clara (January 2021) to the Northern regions of Namibia, later seeded two independent virus introduction events to Zambia in the same month. Since its initial introduction to Namibia, the C.16 lineage was disseminated on numerous occasions between late 2020 and throughout 2021 within the Central and Northern regions of Namibia and was introduced to the Southern regions of Namibia in February 2021. In January 2021, the C.16 lineage was exported to Kimberley, South Africa, where it was disseminated to other regions of the Northern Cape and Gauteng during the same month.

The phylogeography analyses indicated introductions to other neighboring countries. A single introduction event originating from Luanda to the KwaZulu‐Natal province of South Africa was inferred to have occurred during mid‐March 2021 (Figure 4). Similarly, in January 2021, two introductions occurred independently originating from Luanda to the Republic of Congo and the DRC. The latter seeded introductions to Kenya in January 2021.

## 4 DISCUSSION

Comparable to neighboring countries, Angola had endured four waves of SARS‐CoV‐2 infection during the first 27 months of the COVID‐19 pandemic (Figure 1). These waves resulted in 100,000 cases and 2000 deaths, notably fewer than was reported for other African countries (Namibia 158,000, Botswana 305,000, or Zambia 318,000; April 22, 2022^1^). This disparity may reflect lowered rates of testing and the effective containment strategies implemented during the onset of the pandemic. However, despite these reduced case numbers, infections were reported from each of the 18 provinces of Angola. Additionally, underreporting of COVID‐19 deaths remains a matter of great concern in many African countries; these concerns also hold for Angola.^3^

Angolan genome surveillance initiatives identified VOCs, VOIs, and variants under investigation (VUIs) circulating during the study period (Figure 2). These included the first Wuhan variant introduced in March 2020; the five VOCs Alpha, Beta, Delta, Gamma, and Omicron; the VOIs Eta, Theta, Lambda, Mu, and Vui; and other variants such the B.1 and associated sub‐lineages, B.1.1.275, and the C.16 lineage. The results of our study showed that the variants Alpha, Beta, C.16, and Delta were associated with high incidences of community transmission. A notable case of community transmission occurred on a Paenal oil rig, which is located off the coast of Angola, exposing hundreds of workers to COVID‐19, and causing the temporary suspension of the operations of this rig. Our analyses indicated that the B.1.1.275 variant fueled this outbreak. This outbreak resulted in the government‐mandated implementation of health and safety protocols for all oil and gas company workers, such as mandatory testing and isolation for infected individuals.^22^

Distinct genetic combinations fueled local infectious waves in Angola (Figure 2). In contrast to the other African trends,^21^ the first two Angolan waves of COVID‐19 were fueled by an admixture of co‐circulated variants. These included the Alpha, Beta, Delta, Eta, A lineages, B.1 sub‐lineages, B.1.275 and C.16 variants during the first wave, and the Beta, Delta, B.1 and associated sub‐lineages, and C.16 lineages during the third wave. In contrast, the fourth wave was dominated by the VOCs Delta and Omicron. The VOCs Beta, Delta, and Omicron variants were the most prevalent variants sequenced from Angolan samples. A notable finding in this study included the prolonged circulation of the C.16 lineage (Figures 2 and S1), suggesting increased transmissibility compared to the other lineages circulating at the time. This enhanced transmissibility was thought to be due to the D614G and L452R mutations in the Spike gene regions.^23^ The evolutionary success of C.16 was further evident from its international prevalence and was detected in more than 20 countries, of which large numbers of Portuguese samples were noted from December 2020 to March 2021. The C.16 lineage was further detected on multiple occasions during routine screening and genome surveillance of international passengers that tested positive for COVID‐19 upon their return to Angola between June 2020 and February 2021.^24^ The results of this study show that C.16 lineage was exported to neighboring countries and circulated extensively within Namibia.

Epidemiological analyses indicated that Luanda, the capital, and most populous city in Angola, had the highest number of reported cases in the country (Figure 1C). Moreover, samples subjected to whole‐genome sequencing investigations also originated from Luanda. This finding might reflect increased testing and screening implemented at Angola's largest international airport Quatro de Fevereiro, as part of the country's disease mitigation strategies. Luanda remains more vulnerable to high levels of the introduction of new variants as it represents an important center for trade and international travel via air and sea. These variants can become amplified via community transmission due to the dense population of Luanda city.

Despite disease mitigation strategies implemented throughout the pandemic, Angola remained vulnerable to COVID‐19 virus movement across its borders. The introduction of new variants was partially due to the geographic location of Angola and its economic importance as a travel and trade hub for the western regions of Africa. Angola further shares borders with numerous neighboring countries (Figure 1C). Indeed, our analyses indicated that high numbers of virus importation and exportation events occurred between Angola and the Republic of Congo, the DRC, to its North, Zambia to the East, and Namibia to the South. Policing these borders poses logistical challenges as these large geographical regions include forested regions, savannahs, and other natural barriers^25^ that impede effective border control and monitoring. It, therefore, results in porous borders with a high frequency of illicit movement of people. Indeed, Angola was deemed one of the top countries harboring migrant populations in Africa.^26^

Our results indicated virus transmission between Angola, neighboring countries, and countries further abroad. The large‐scale virus importation and exportation events between Angola and Portugal noted in this study most likely reflect their strong historical and cultural histories, as Angola represents a former Portuguese colony.^27^ As a result, there was significant travel between the two countries, with many people from Angola traveling to Portugal for work, study, or tourism. In agreement with these findings, the first reported case of COVID‐19 was sampled from a traveler returning from Portugal (Figure 1A). Our analyses further indicated a strong virus movement between Angola and Brazil. Besides cultural and linguistic similarities, strong ancestral ties remain, reflecting the strong ties springing from colonial era where the relocation of Angolan slaves was brought to Brazil.^28^ Besides the cultural and historical ties, these countries also share strong economic and business interests.

Our analyses indicated that the first introduction event was inferred during the first months of 2020, and most likely originated from Southern African countries, despite the first reported case being linked to Portugal (Figure 1A). In agreement with previous findings,^29^ our analyses indicated that cases may have occurred undetected before the first case was reported. Similar to Brazil and Portugal, the high level of virus introduction and exportation events noted between Angola, Namibia, and South Africa were most likely due to their strong economic ties, particularly in the mining and construction sectors, and many Angolans travel to South Africa and Namibia for business, education, and tourism purposes.^25^ However, the high number of inferred importation events originating from South Africa may be an artifact of South Africa's higher testing and enhanced genome surveillance capacity.^30^, ^31^ Therefore, the epidemiological linkage between Angola and other neighboring countries, apart from South Africa, may have occurred as a result of limited testing in these regions, and may therefore not have been detected in our analyses.

The high number of inferred virus exchange events reported in this study emphasized the global importance of effective genome surveillance in Angola. Despite Angola's limited surveillance capacity and infrastructure, Angolan genome surveillance identified the prevalence of the VOCs, VOIs, and VUI, C.16, and B.1.1.275 (Figure 2A). These efforts further detected the presence of the Omicron lineage days following its first report from Southern Africa, indicating improved and effective genome surveillance as the pandemic progressed.

Despite these achievements in genomic surveillance, the informativeness of this study was limited due to the disparity in data in Angola's surveillance initiative. No sequencing was performed before June 2020 and no isolates were sampled from Eastern provinces such as Lunda Norte, Lunda Sul, Moxico, and the western province, Benguela (Figure 1C). This is most likely due to the need for Angola to rely on external, out‐of‐country laboratories for sequencing as no large‐scale in‐country facilities were not available during the peak of the pandemic. Outsourcing genome surveillance is often associated with high costs. Moreover, effective cold chain management and referral systems that provide samples for genome sequencing may also not be optimized, limiting genome surveillance initiatives. This is particularly concerning as real‐time data generation is of fundamental importance to guide pandemic mitigation initiatives. A further limitation of this study was the uneven sampling that occurred during the study period. The paucity in the availability of samples reflected periods of high infectious waves in the country and the sampling was dependent on reference laboratories submitting samples for sequencing. The limited availability of samples throughout the pandemic hampers not only real‐time data surveillance but also the study of pandemic progression in the country.

Beyond the COVID‐19 pandemic, genome surveillance may in the future aid disease mitigation of other disease outbreaks. Indeed, several disease outbreaks in Angola were recently reported; suggesting that Angola may remain vulnerable to future outbreaks. These include the largest recorded outbreak of the Marburg virus occurring in 2004–2005,^24^ which began in the province of Uíge in northern Angola and quickly spread to other parts of the country. Also, a large outbreak of yellow fever that occurred during 2015–2016 in the northern and central parts of the country,^32^ cholera outbreaks reported between 2016 and 2019,^33^ and a recent measles outbreak during 2019 and 2020.^34^ These outbreaks emphasize the need for enhanced pathogen and genome surveillance within these regions. A robust pathogen genomic surveillance infrastructure remains of global importance to curb the dissemination of emerging infectious diseases.

The COVID‐19 pandemic further highlighted several weaknesses in the country's pandemic response despite the disease mitigation strategies. This included the strain placed on the country's already struggling healthcare system.^6^ Angola had limited resources and a shortage of healthcare workers during the peak of the pandemic. Healthcare workers were particularly vulnerable during the peak of the pandemic,^35^ due to shortages of personal protective equipment and other essential medical supplies in healthcare settings. The procurement of vaccines and the distribution were initially slow and there were concerns about the government's ability to effectively manage the vaccination campaign. Moreover, large‐scale vaccine hesitancy was due to a lack of trust in the healthcare system and government institutions. Additionally, access to clean water and sanitation in rural regions remains a concern.^36^

## 5 CONCLUSIONS

With a focus on the occurrence of VOCs, VOIs, and VUMs, our findings offer a thorough overview of the genomic epidemiology of SARS‐CoV‐2 in Angola during the first 27 months, and the peak of the COVID‐19 pandemic. According to the data generated in this study, southern Africa seeded Angola's first import of the SARS‐CoV‐2 virus. The results of this study showed that several virus exchanges occurred between Namibia, Brazil, Portugal, South Africa, and Angola. These findings illustrate how trade, commerce, and historical relationships contribute to the transmission of pathogens within the country. Isolates that were particularly sequenced for this study served as an example of community transmission in Angola, including outbreaks occurring in high‐risk settings such as oil rigs. Therefore, to better inform policies and actions to combat COVID‐19, our work underscores the importance of continued genomic surveillance in Angola. However, a lack of a robust genome surveillance network within Angola and a strong dependence on out‐of‐country sequencing facilities limit real‐time data generation. This in turn hampers timely disease outbreak responses that remain of the utmost importance to mitigate the ongoing COVID‐19 pandemic and future emerging and re‐emerging diseases.

## AUTHOR CONTRIBUTIONS

**Ngiambudulu M. Francisco**: Conceptualization; methodology; writing; review and editing; funding acquisition; supervision. **Stephanie van Wyk**: Methodology; investigation; writing—original draft; review and editing; visualization. **Monika Moir**: Methodology; investigation; visualization. **James Emmanuel San**: Methodology; investigation; review and editing; visualization. **Cruz S. Sebastião**: Methodology; investigation. **Houriiyah Tegally**: Methodology; investigation; review and editing; visualization. **Joicymara Xavier**: Methodology; investigation; review and editing; visualization. **Akhil Maharaj**: Investigation. **Zoraima Neto**: Investigation. **Pedro Afonso**: Investigation. **Domingos Jandondo**: Investigation. **Joana Paixão**: Investigation. **Julio Miranda**: Investigation. **Kumbelembe David**: Investigation. **Luzia Inglês**: Investigation. **Amilton Pereira**: Investigation. **Agostinho Paulo**: Investigation. **Raisa Rivas Carralero**: Investigation. **Helga Reis Freitas**: Investigation. **Franco Mufinda**: Investigation. **Silvia Lutucuta**: Investigation. **Mahan Ghafari**: Investigation. **Marta Giovanetti**: Investigation. **Jennifer Giandhari**: Investigation. **Sureshnee Pillay**: Investigation. **Yeshnee Naidoo**: Investigation. **Lavanya Singh**: Investigation. **Derek Tshiabuila**: Investigation; methodology. **Darren Patrick Martin**: Investigation; methodology. **Lucious Chabuka**: Investigation. **Wonderful Choga**: Investigation. **Dorcas Wanjohi**: Investigation. **Sarah Mwangi**: Investigation. **Yusasha Pillay**: Investigation. **Yenew Kebede**: Investigation. **Edwin Shumba**: Investigation. **Pascale Ondoa**: Investigation. **Cheryl Baxter**: Investigation; supervision. **Eduan Wilkinson**: Methodology; investigation; review and editing; supervision. **Sofonias Kifle Tessema**: Project administration. **Aris Katzourakis**: Investigation; methodology. **Richard Lessells**; Investigation; project administration; supervision. **Tulio de Oliveira**: Funding acquisition; project administration; supervision. **Joana Morais**: Funding acquisition; project administration.

## CONFLICT OF INTEREST STATEMENT

The authors declare no conflicts of interest.

### PEER REVIEW

The peer review history for this article is available at https://www.webofscience.com/api/gateway/wos/peer-review/10.1111/irv.13198.

## Supporting information


**Figure S1.** Illustration of the proportions of SARS‐CoV‐2 lineages in the Republic of Angola per month from June 2020 to February 2022.
**Figure S2.** The mean monthly viral importation events into Angola and exports from Angola (shown in gray), standard deviation displayed with shading around the line. The mean importation (A) and exportation (B) events for the three countries with the greatest number of viral exchanges with Angola are also displayed.

## Data Availability

All data produced in the present study are available upon reasonable request to the corresponding author.
