# Supplementary material for: Insights into SARS‐CoV‐2 in Angola during the COVID‐19 peak: Molecular epidemiology and genome surveillance
Source: Influenza Other Respir Viruses. 2023 Sep 22;17(9):e13198. doi: 10.1111/irv.13198 (PMC10515134; doi:10.1111/irv.13198)
Supplement: Supplementary file 1 — Figure S1. Illustration of the proportions of SARS‐CoV‐2 lineages in the Republic of Angola per month from June 2020 to February 2022. Figure S2. The mean monthly viral importation events into Angola and exports from Angola (shown in gray), standard deviation displayed with shading around the line. The mean importation (A) and exportation (B) events for the three countries with the greatest number of viral exchanges with Angola are also displayed. [file IRV-17-e13198-s001.docx]

**Supplementary files** **(Supplementary figures)**


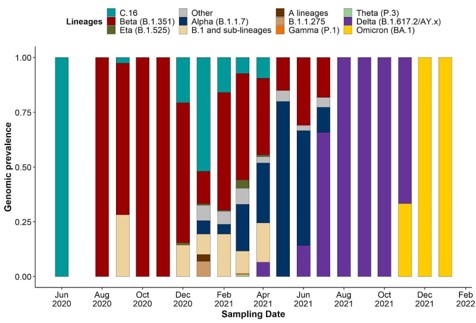


**Supplementary Figure 1**: Illustration of the proportions of SARS-CoV-2 lineages in the Republic of Angola per month from June 2020 to February 2022.


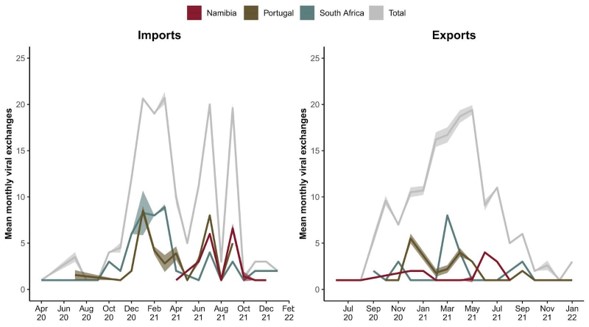


**Supplementary Figure 2:** The mean monthly viral importation events into Angola and exports from Angola (shown in grey), standard deviation displayed with shading around the line. The mean importation (A) and exportation (B) events for the three countries with the greatest number of viral exchanges with Angola are also displayed.
